# Teriflunomide restores 5-azacytidine sensitivity via activation of pyrimidine salvage in 5-azacytidine-resistant leukemia cells

**DOI:** 10.18632/oncotarget.19436

**Published:** 2017-07-22

**Authors:** Satoshi Imanishi, Ryoko Takahashi, Seiichiro Katagiri, Chiaki Kobayashi, Tomohiro Umezu, Kazuma Ohyashiki, Junko H. Ohyashiki

**Affiliations:** ^1^ Department of Molecular Oncology, Institute for Medical Science, Tokyo Medical University, Nishi-Shinjuku, Shinjuku, Tokyo, Japan; ^2^ Department of Hematology, Tokyo Medical University, Nishi-Shinjuku, Shinjuku, Tokyo, Japan

**Keywords:** azacytidine, teriflunomide, pyrimidine metabolism, sensitization

## Abstract

Previous studies showed that downregulation of pyrimidine salvage underlies resistance against 5-azacytidine (AZA), indicating an important role for *de novo* pyrimidine synthesis in AZA resistance. Because *de novo* pyrimidine synthesis is inhibited by the immunomodulator teriflunomide and its pro-drug leflunomide, we examined the effect of combined treatment with AZA and teriflunomide on AZA resistance to develop a novel strategy to cancel and prevent AZA resistance. Teriflunomide markedly inhibited the growth of AZA-resistant human leukemia cell lines (R-U937 and R-HL-60) in comparison with their AZA-sensitive counterparts (U937 and HL-60). In the presence of a non-toxic concentration of teriflunomide (1 μM), AZA induced apoptosis in AZA-resistant cells and leukemia cells from AZA-resistant patients. AZA acted as a DNA methyltransferase 3A inhibitor in AZA-resistant cells in the presence of 1 μM teriflunomide. Although AZA-sensitive cells acquired AZA resistance after continuous treatment with AZA for 42 days, the growth of AZA-sensitive cells continuously treated with the combination of AZA and teriflunomide was significantly inhibited in the presence of AZA, demonstrating that the combined treatment prevented AZA resistance. These results suggest that combined treatment with AZA and teriflunomide can be a novel strategy to overcome AZA resistance.

## INTRODUCTION

5-azacytidine (AZA) is approved for the treatment of patients with myelodysplastic syndrome (MDS) in Europe, the United States, Japan, and other countries. AZA yields a 40–60% response rate in these patients [1]. However, some patients treated with AZA develop resistance to the drug after various treatment durations [2, 3]. Although the prognosis of MDS patients after AZA treatment failure is poor, with a median overall survival time of 5.6 months [4], a strategy to cancel resistance against AZA has not been developed.

AZA taken up by cells is mono-phosphorylated by uridine-cytidine kinase (UCK) 1 or UCK2, subsequently phosphorylated by other enzymes to produce 5-AZA-diphosphate (azaCDP) and 5-AZA-triphosphate, and incorporated into RNA. Part of azaCDP is converted to 2′-deoxy-5-AZA-triphosphate (d-azaCTP) by ribonucleotide reductase [5]. d-azaCTP is incorporated into DNA and forms irreversible DNA methyltransferase (DNMT)/DNA complexes. Removal of DNMT/DNA complexes and repair of DNA result in degradation of DNMT proteins accompanying demethylation of DNA [6].

Previously, we reported that AZA resistance in R-U937 cells and R-HL-60 cells (AZA-resistant cells), which we originally created from U937 cells and HL-60 cells, respectively [7], involves downregulation of *UCK2* expression and conversion of UTP to CTP. Valencia et al. reported that lower expression of *UCK1* mRNA correlates to primary resistance against AZA in patients with MDS [8]. Because of the roles of UCK1 and UCK2 in pyrimidine salvage, these findings indicate the important role of the alternative pyrimidine-supplying mechanism—*de novo* pyrimidine synthesis—in AZA resistance in cell lines and patients. It is well established that *de novo* pyrimidine synthesis is inhibited by teriflunomide and its pro-drug leflunomide, which are the immunomodulators approved for the treatment of multiple sclerosis and rheumatoid arthritis, respectively [9, 10]. Therefore, we examined the effect of combined treatment of AZA and teriflunomide on AZA sensitivity and resistance.

## RESULTS

### Teriflunomide-activated pyrimidine salvage in AZA-resistant cells

**Figure 1:**
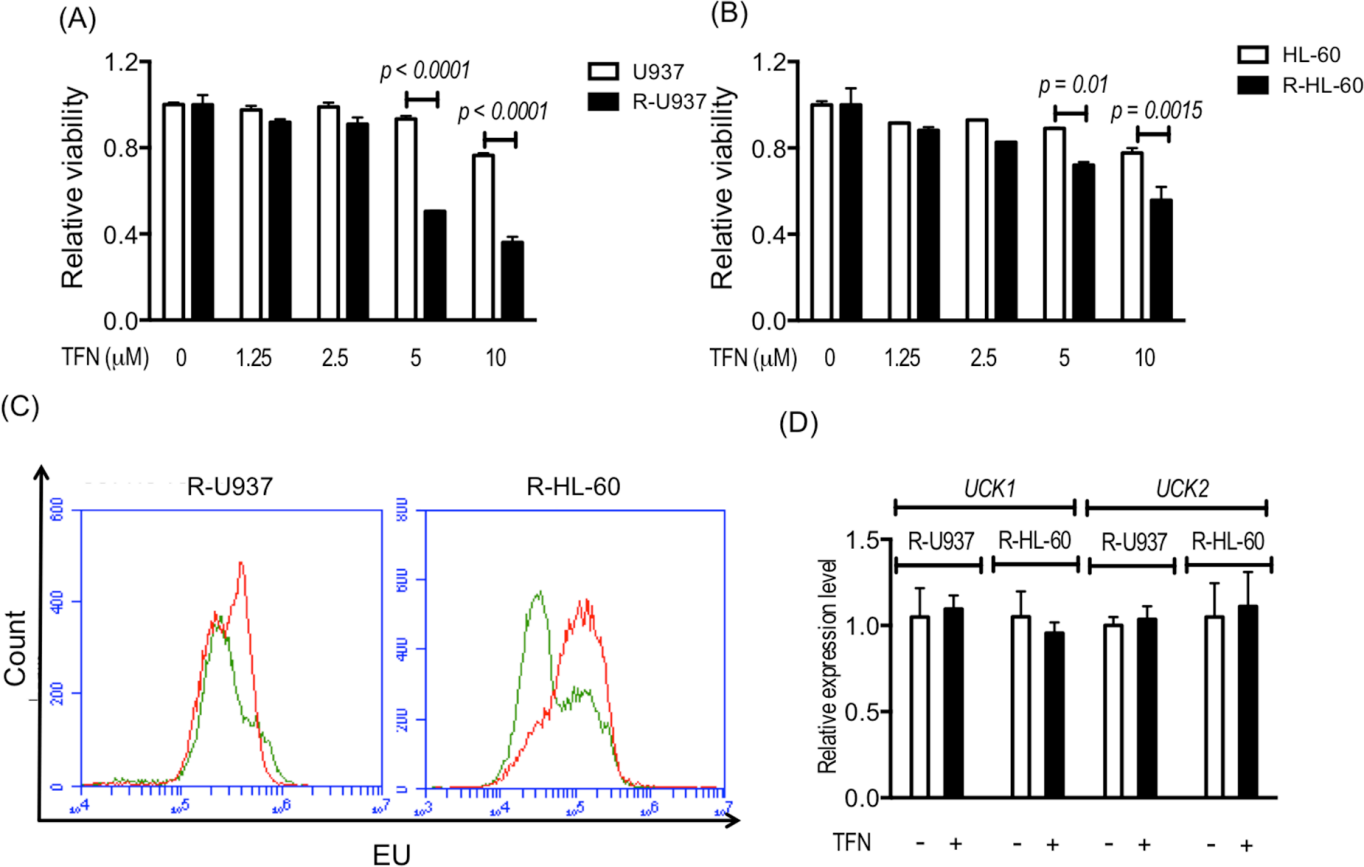
Viability of U937 cells and R-U937 cells **(A)** and of HL-60 cells and R-HL-60 cells **(B)** after treatment with teriflunomide (TFN) for 72 h. The *p*-values are indicated. *N.S.* indicates nonsignificant results of the *t*-test following two-way ANOVA. The results of three independent experiments are shown. **(C)** Analysis of EU availability in R-U937 cells (left panel) and R-HL-cells (right panel) without and with 1 μM TFN treatment for 72 h. Green lines: without treatment; red lines: with treatment. **(D)** Relative mRNA expression levels of UCK1 and UCK2 normalized with the mRNA expression level of ACTB in R-U937 and R-HL-60 cells without and with 1 μM TFN treatment for 72 h. The results of three independent experiments are shown.

After treatment with teriflunomide at a dose equivalent to the serum concentration of teriflunomide (1.25–10 μM) in patients with rheumatoid arthritis treated with leflunomide, viability of R-U937 and R-HL-60 cells was significantly decreased in comparison with their AZA-sensitive counterparts (U937 and HL-60 cells; Figure 1A, 1B). Treatment with 1 μM teriflunomide notably increased the fraction with a higher signal for 5-ethynyluridine (EU), which is phosphorylated via pyrimidine salvage and is incorporated with RNA [11] in both types of AZA-resistant cells (Figure 1C), whereas the mRNA expression of *UCK1* and *UCK2* was not affected by the presence of teriflunomide (Figure 1D).

### AZA-induced apoptosis in AZA-resistant cells in the presence of teriflunomide

**Figure 2:**
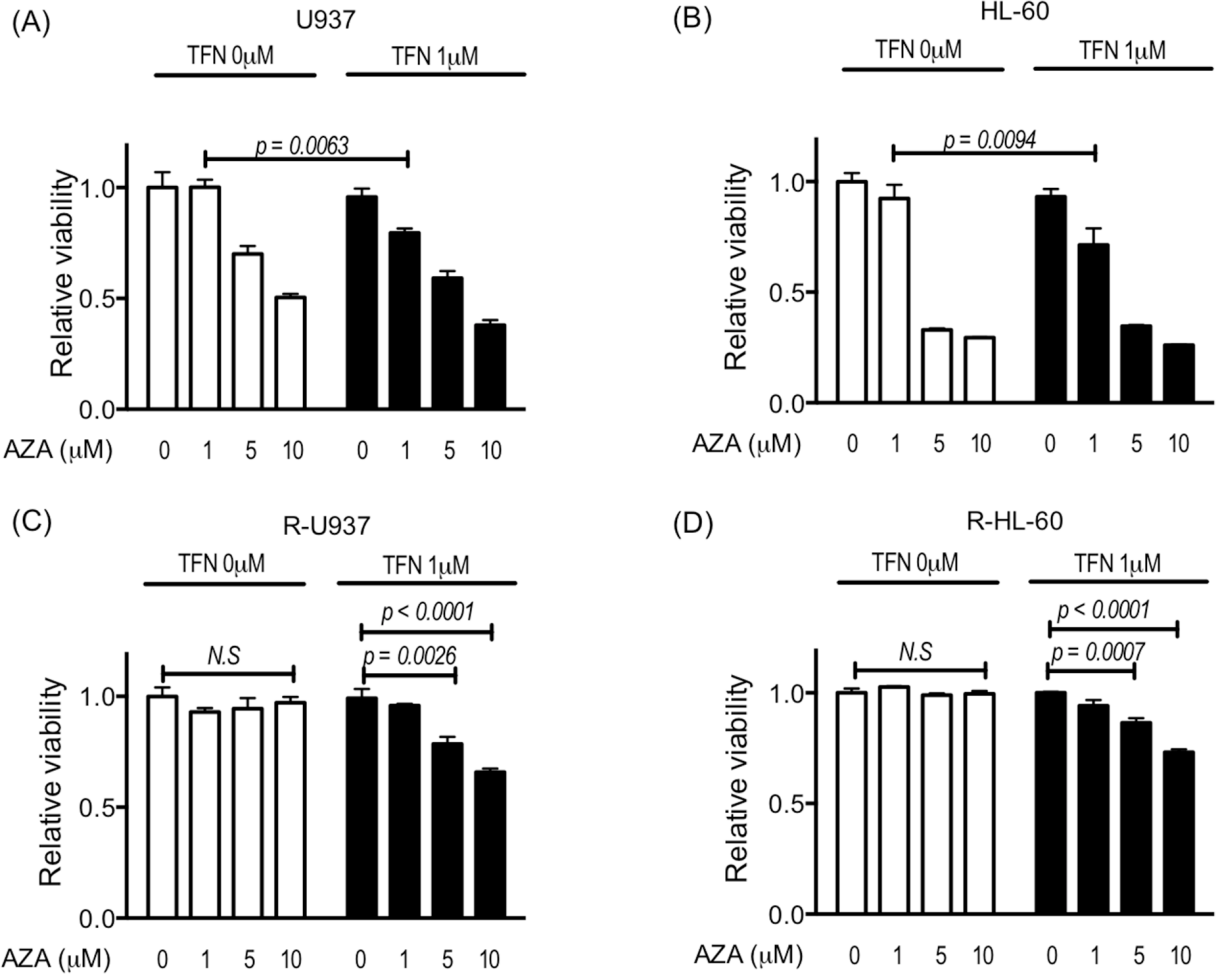
Viability of U937 cells **(A),** HL-60 cells **(B)**, R-U937 cells **(C)**, and R-HL-60 cells **(D)** after treatment with AZA (0, 1, 5, 10 μM) for 72 h in the absence or presence of 1 μM teriflunomide (TFN). The *p*-values of significance are indicated. *N.S.* indicates nonsignificant results of the *t*-test and two-way ANOVA. The results of three independent experiments are shown.

Next, we examined the effect of teriflunomide on the growth inhibitory activity of 1, 5, and 10 μM AZA, which are the relevant doses to the serum concentration of AZA in MDS patients [12, 13]. Although treatment with teriflunomide up to 1.25 μM for 72 h did not show significant growth-inhibitory effects in the cells used in this study (Figure 1), 1 μM teriflunomide enhanced the cytotoxic effects of 1 μM AZA in U937 and HL-60 cells (Figure 2A, 2B). In R-U937 and R-HL-60 cells, we detected a significant decrease in the viability in an AZA dose-dependent manner in the presence of 1 μM teriflunomide (Figure 2C, 2D), demonstrating that the presence of teriflunomide restored AZA sensitivity in AZA-resistant cells.

To examine whether the effects of the combined treatment with AZA and teriflunomide was the true synergic effect, we calculated combined index of 25% inhibition of cell growth (CI 25) using the Chou-Talalay method [14]. CI 25 of the combined treatment with AZA and teriflunomide was 0.72 for U937 cells, 0.48 for HL-60 cells, 0.54 for R-U937 cells, and 0.47 for R-HL-60 cells, suggesting that the effect of the combined treatment with AZA and teriflunomide was the true synergic effect.

**Figure 3:**
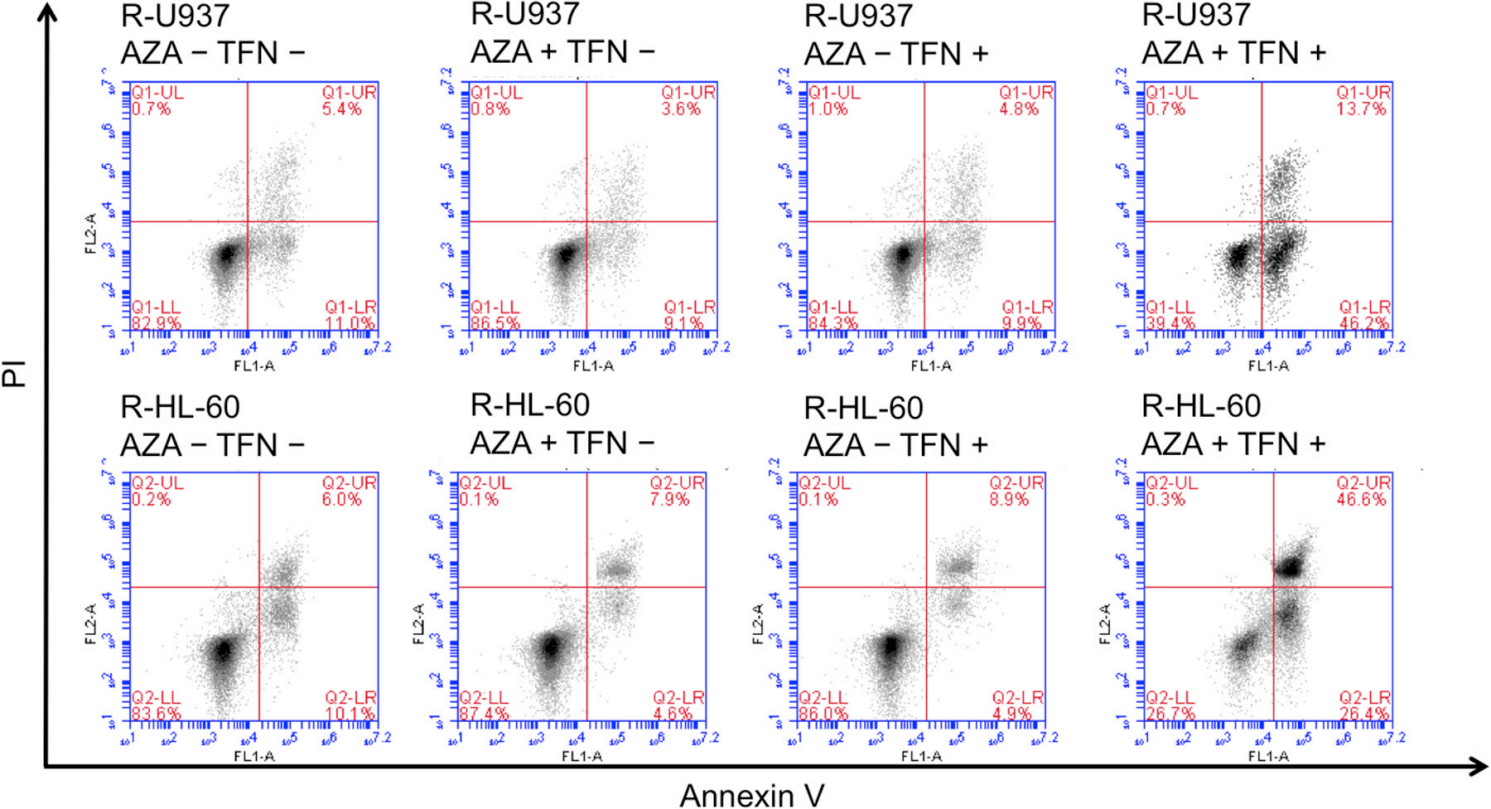
Analysis of apoptosis in R-U937 cells. (upper panels) and R-HL-60 cells (lower panels) of mock treatment, treatment with 10 μM AZA alone, treatment with 1 μM teriflunomide (TFN) alone, and combination treatment with AZA and TFN for 96 h.

In the analyses of annexin V-propidium iodide double staining, we detected induction of apoptosis in AZA-resistant cells after combined treatment for 96 h in R-U937 cells (Figure 3, upper panels) and R-HL-60 cells (Figure 3, lower panels), but not in those treated with AZA or teriflunomide alone, implying that the AZA-dependent decrease of viability in AZA-resistant cells after combined treatment was accompanied by apoptosis.

**Figure 4:**
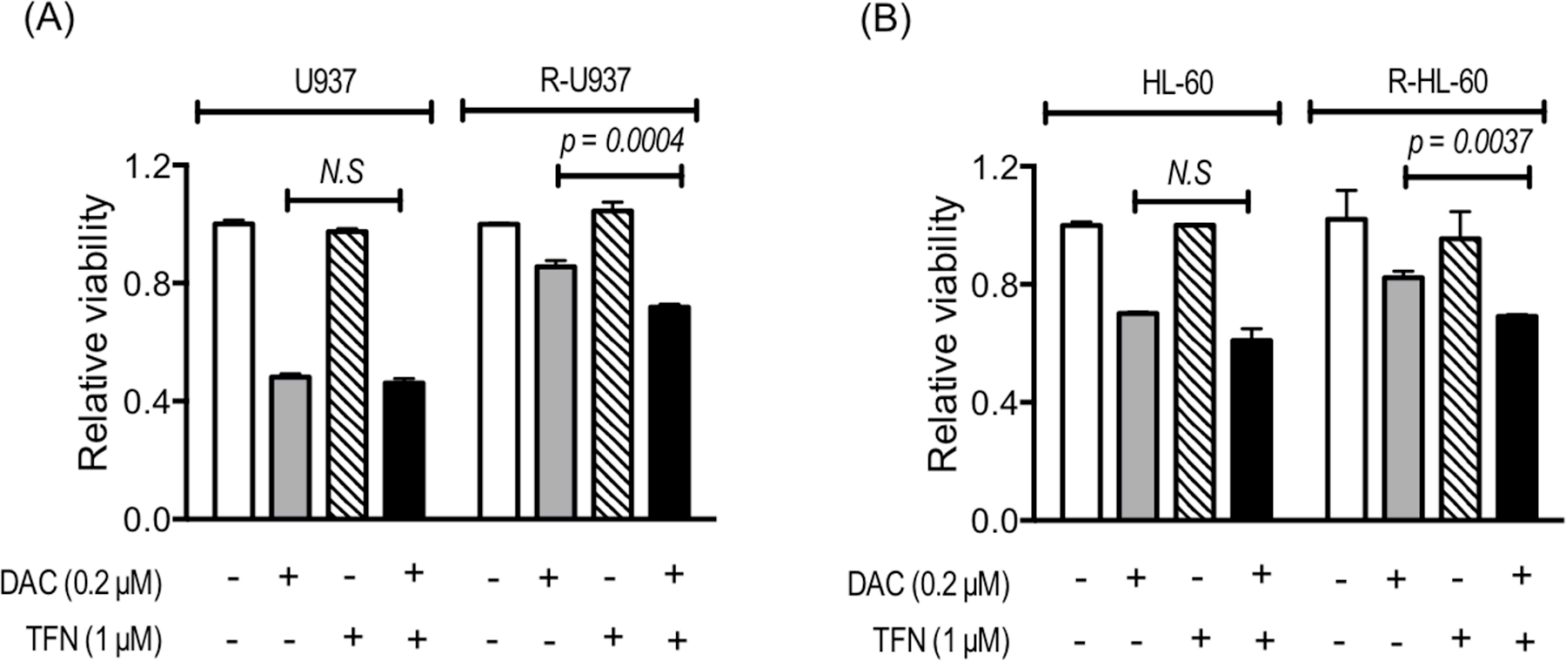
Viability of U937 and R-U937 cells **(A)** and HL-60 and R-HL-60 cells **(B)** after treatment with 0.2 μM DAC for 72 h in the absence or presence of 1 μM teriflunomide (TFN). The results of three independent experiments are shown.

Because R-U937 and R-HL-60 cells showed lower sensitivity to decitabine (5-aza-2'-deoxycytidine: DAC) in comparison with their AZA-sensitive counterparts [7], we examined the effects of treatment with teriflunomide on DAC sensitivity. The presence of 1 μM teriflunomide did not affect the viability of U937 and HL-60 cells cultured with 0.2 μM DAC for 72 h, whereas greater growth repression by 0.2 μM DAC in the presence of 1 μM teriflunomide was observed in R-U937 and R-HL-60 cells (Figure 4A, 4B).

### AZA acted as a DNMT inhibitor in AZA-resistant cells in the presence of teriflunomide

**Figure 5:**
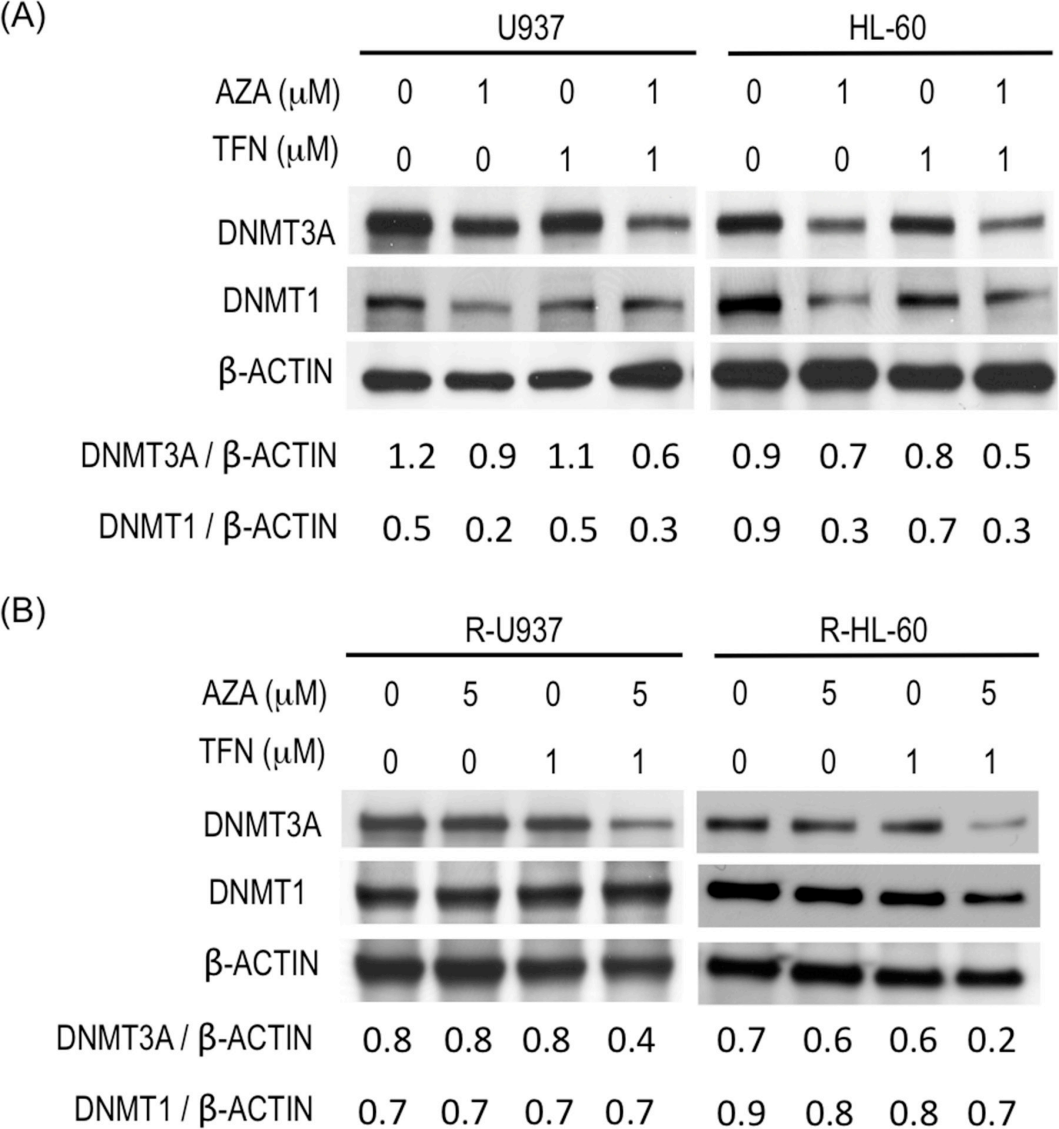
**(A)** Amounts of DNMT3A and DNMT1 in U937 cells and HL-60 cells after treatment with the indicated agent for 48 h. Values indicate the relative amounts of DNMT3A and DNMT1 normalized with β-ACTIN. **(B)** Amounts of DNMT3A and DNMT1 in R-U937 and R-HL-60 cells after treatment with the indicated agent for 48 h. Values indicate the relative amounts of DNMT3A and DNMT1 normalized with β-ACTIN.

To clarify whether AZA acted as a DNMT inhibitor in AZA-resistant cells in the presence of teriflunomide, we examined DNMT3A and DNMT1 protein amounts. (DNMT3B was not examined because its expression was not detected in R-U937 and R-HL-60 cells in our previous study [7].) In AZA-sensitive cells, treatment with 1 μM AZA for 48 h decreased DNMT1 and DNMT3A proteins and the presence of 1 μM teriflunomide enhanced degradation of DNMT3A, but not DNMT1, by AZA treatment (Figure 5A). The effects of treatment with 1 μM teriflunomide alone on these proteins were not significant. Combined treatment with 5 μM AZA and 1 μM teriflunomide for 48 h significantly decreased the amount of DNMT3A protein in AZA-resistant cells, whereas the amount of DNMT3A was not affected when these cells were treated with 5 μM AZA or 1 μM teriflunomide alone (Figure 5B). The amount of DNMT1 protein did not change after treatment with each agent or their combination.

To examine why the amount of DNMT1 did not change after the combined treatment, we compared DNA amount in between AZA-resistant and AZA-sensitive cells by using a chromatin immunoprecipitation (ChIP) assay with anti-DNMT1 antibody or anti-DNMT3A antibody (Supplementary Figure 1). With anti-DNMT1 antibody, the amount of precipitated DNA from AZA-resistant cells was significantly less than that from AZA-sensitive cells, indicating that recruitment of DNMT1 on DNA is less frequent in AZA-resistant cells. No such significant difference was detected in the experiments using anti-DNMT3A antibody.

### Teriflunomide canceled AZA resistance in leukemia cells obtained from patients

**Figure 6:**
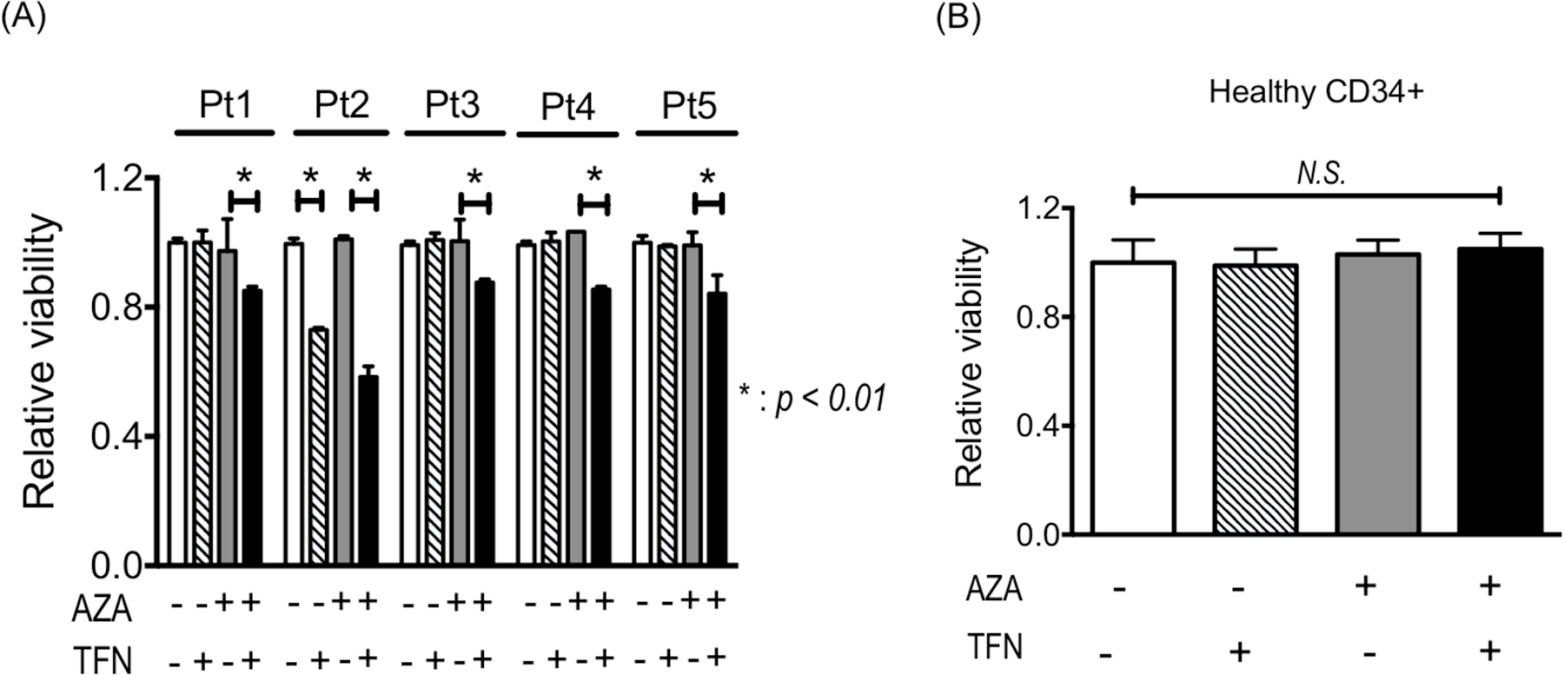
**(A)** Viability of leukemia cells from patients with AZA resistance after treatment with 1 μM AZA and/or 1 μM teriflunomide (TFN) for 24 h. *p < 0.01. **(B)** Viability of healthy CD34^+^ cells after treatment with 1 μM AZA and/or 1 μM TFN for 48 h.

The experiments using cell lines demonstrated that teriflunomide could cancel AZA resistance, so we examined whether AZA resistance in leukemia cells from patients is canceled by combined treatment of AZA with teriflunomide. In leukemia cells with AZA resistance from four patients (nos. 1, 3, 4, 5), decreased viability was detected when they were treated with 1 μM AZA combined with teriflunomide at 1 μM; however, 1 μM AZA and 1 μM teriflunomide individually were not cytotoxic (Figure 6A). Leukemia cells from patient 2 showed relatively higher sensitivity to teriflunomide, resulting in decreased viability after treatment with teriflunomide alone; however, combined treatment reduced their viability much more so (Figure 6A). The viability of CD34-posisitve cells from a healthy donor was not affected by treatment with 1 μM AZA or 1 μM teriflunomide or by the combined treatment, indicating that treatment with AZA and/or teriflunomide did not influence the proliferation of healthy hematopoietic stem/progenitor cells (Figure 6B).

### Teriflunomide prevented AZA-resistance *in vitro*

**Figure 7:**
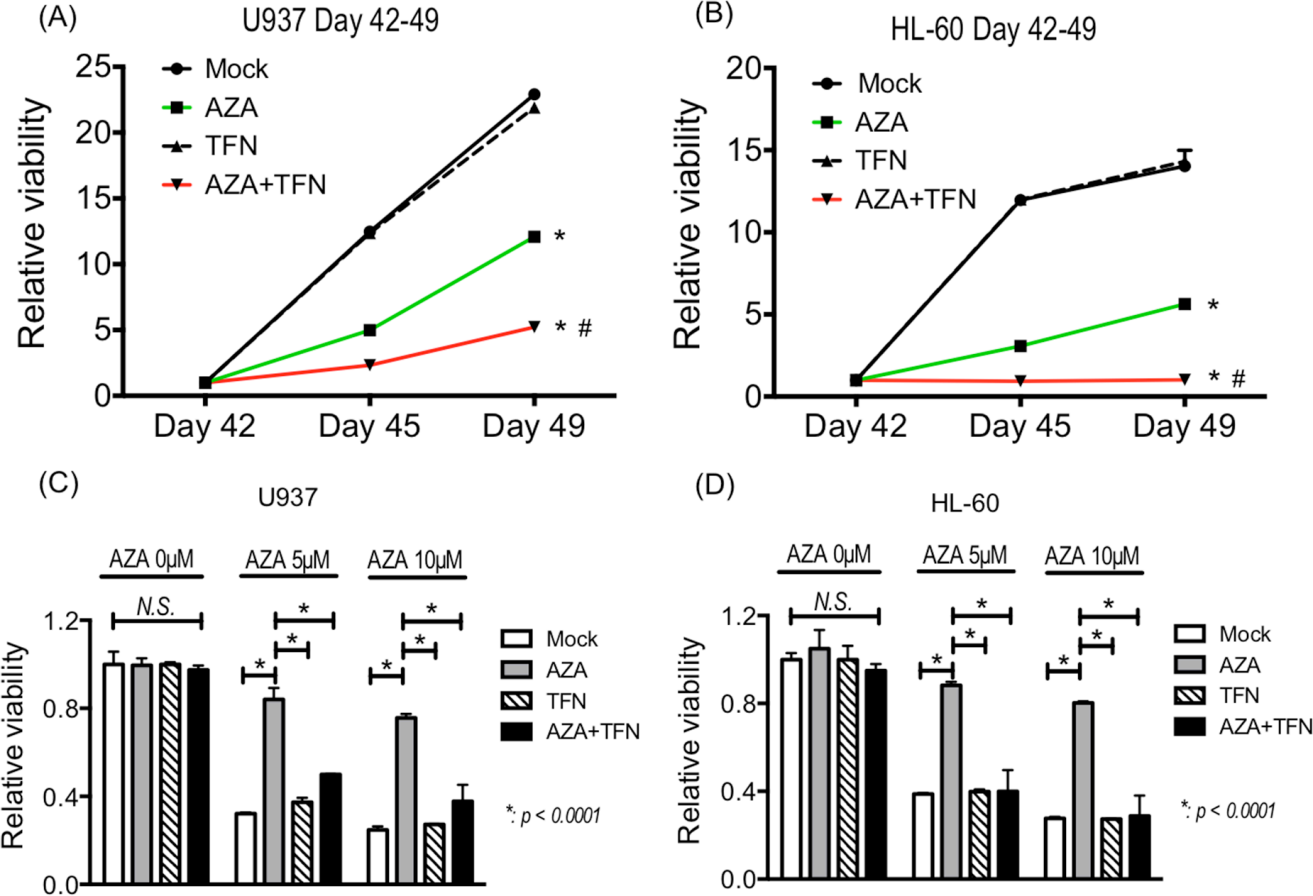
**(A, B)** The growth of U937 cells (A) and HL-60 cells (B) treated with 5 μM AZA and/or 1 μM teriflunomide (TFN) for 42 days. *p < 0.01 in comparison with mock or TFN-treated cells, #*p* < 0.01 in comparison with AZA-treated cells. **(C, D)** The AZA sensitivity of U-937 cells (C) and HL-60 cells (D) treated with 5 μM AZA and/or 1 μM TFN for 42 days.

Finally, we examined whether teriflunomide can prevent the acquisition of AZA resistance in AZA-sensitive cells. After continuous treatment with 5 μM AZA for 42 days, the growth of U937 and HL-60 cells was detected even in the presence of 5 μM AZA, indicating that the cells had acquired AZA resistance. Continuous treatment with 1 μM teriflunomide for 42 days did not affected in the growth of U937 and HL-60 cells, and their growth when treated with a combination of 5 μM AZA and 1 μM teriflunomide for 42 days was significantly slower than that of AZA- or teriflunomide-treated cells (Figure 7A, 7B).

To assess the sensitivity to AZA in U937 and HL-60 cells after continuous treatment, we cultured them in the presence of 5 and 10 μM AZA for 72 h. As expected, U937 and HL-60 cells after continuous treatment with AZA showed lower sensitivity to AZA, whereas these cells after continuous combined treatment showed decreased viability, as did control cells without treatment and cells continuously treated with teriflunomide for 42 days (Figure 7C, 7D). These findings demonstrate that the combined treatment prevented the acquisition of resistance against AZA in U937 and HL-60 cells.

## DISCUSSION

Drug repositioning, which is a pharmaceutical approach to finding novel activity of existing drugs, has become popular because it reduces development costs and accelerates regulatory approval [15]. We found teriflunomide, an immunomodulator approved for treatment of multiple sclerosis, to be an agent that can overcome and prevent AZA resistance in leukemia by using an approach based on the molecular mechanism underlying AZA resistance. Recent attention to drug repositioning has mainly focused on computational approaches using virtual screening of the comprehensive libraries of approved compounds based on three-dimensional structures of target proteins [16–18]. However, mechanism-based drug repositioning may be another approach to finding drugs that can overcome drug resistance in cancer. To date, several molecular-targeted drugs, including HDAC inhibitors, have been used to overcome AZA resistance, but none of them restores the action of AZA in leukemia cells that creates AZA resistance. We demonstrated for the first time that mechanism-based drug repositioning could resensitize AZA-resistant human leukemia cells to AZA.

Despite the fact that downregulated *UCK1* or *UCK2* expression in AZA resistance has been extensively studied [7, 8], the role of *de novo* pyrimidine synthesis in AZA resistance is not fully understood. In this study, we demonstrated that sensitivity to teriflunomide in AZA-resistant cells was higher than that in their AZA-sensitive counterparts, suggesting that AZA-resistant cells more strongly depend on *de novo* pyrimidine synthesis. Treatment with teriflunomide at a non-cytotoxic dose increased the availability of EU in AZA-resistant cells, but it did not induce re-expression of *UCK1* or *UCK2*. Therefore, inhibition of *de novo* pyrimidine synthesis might activate pyrimidine salvage without induction of *UCK1* or *UCK2*. Teriflunomide might reduce the pool size of CTP and dCTP and thus decrease competition for the incorporation of azaCTP into RNA and d-azaCTP into DNA. It is noteworthy that AZA also inhibited cell growth in the presence of teriflunomide in freshly obtained leukemia cells from patients with AZA resistance. Therefore, inhibition of *de novo* pyrimidine synthesis by teriflunomide may play a key role in the restoration of AZA sensitivity in freshly obtained leukemia cells from patients as well as cell lines.

Another important finding is the notable decrease of DNMT3A in AZA-resistant cells after combined treatment with AZA and teriflunomide. As the ChIP assay demonstrated, the recruitment of DNMT1 on DNA was less frequent in AZA-resistant cells than AZA-sensitive counterparts. Given the hypomethylation of DNA in AZA-resistant cells [7], DNMT3A might be recruited on DNA more frequently than DNMT1 in AZA-resistant cells. The decrease of DNMT3A might indicate that AZA acted as a DNMT inhibitor in AZA-resistant cells in the presence of teriflunomide. Therefore, combined treatment with AZA and teriflunomide or leflunomide could be a promising strategy to sensitize patients with MDS or acute myeloid leukemia (AML) who developed AZA resistance. In addition to restoration of AZA sensitivity in AZA-resistant cells, enhancement of growth inhibitory activity of AZA at 1 μM accompanied with increase of degradation of DNMT3A but not DNMT1 was found in AZA-sensitive cells. Although the reason why teriflunomide did not increase the degradation of DNMT1 remains unclear, one possibility is that DNMT1 recruited on DNA in AZA-sensitive cells might be nearly exhausted in the presence of 1 μM AZA.

The cross-resistance against DAC could be weakened in the presence of teriflunomide in R-U937 and R-HL-60 cells, suggesting that the combined treatment with DAC and teriflunomide might be another treatment choice for myeloid neoplasm with AZA resistance. On the other hand, inhibition of *de novo* pyrimidine synthesis by teriflunomide failed to enhance the growth inhibitory activity of DAC in AZA-sensitive cells. Unlike our result, Raynal *et al.* demonstrated that inhibition of *de novo* pyrimidine synthesis by an inhibitor of CTP synthase enhances the cytotoxic activity of DAC by enhancing the incorporation of DAC into DNA [19]. Despite the fact that the sensitivity to DAC might correlate with de novo pyrimidine synthesis as well as AZA sensitivity, the key enzyme determining the sensitivity to AZA and DAC might be different. More detailed research on the role of *de novo* pyrimidine synthesis in the sensitivity to AZA and DAC is needed.

Evidence suggests that AZA resistance requires a shift in the pyrimidine-supplying pathway from salvage-dependent to *de novo* synthesis-dependent [7, 20]. In the current study, we demonstrated that teriflunomide prevented the acquisition of AZA resistance. This prevention might be accompanied with maintenance of pyrimidine salvage activity through inhibition of *de novo* pyrimidine synthesis. The combined treatment with AZA and teriflunomide could be a novel strategy to prevent the development of AZA resistance.

Some limitations exist in our approach. First, our results should be confirmed by *in vivo* studies using animal model. Similarly, we could not examine whether teriflunomide or leflunomide may improve the immunological environment of patients with MDS in our *in vitro* study, but the immunomodulating activity of teriflunomide and leflunomide is well established [9, 10].

In conclusion, we provide evidence of the importance of *de novo* pyrimidine synthesis in AZA resistance and of its potential as a therapeutic target of *de novo* pyrimidine synthesis to overcome and prevent AZA resistance. Anti-cancer activity of teriflunomide has been reported for various tumors [21–23], including hematologic malignancies [24]. Therefore, not only combined use with AZA but also use of a higher dose of teriflunomide might be a potent strategy for the treatment of AML with AZA resistance. However, some questions still remain, such as whether *de novo* pyrimidine synthesis inhibitors can sensitize primary resistance against AZA and whether *de novo* pyrimidine synthesis inhibitors can prevent the development of DAC resistance. To answer these questions, further studies are necessary.

## MATERIALS AND METHODS

### Cells and reagents

U937 and HL-60 cells were purchased from ATCC (Manassas, VA, USA). AZA-resistant cell lines (R-U937 and R-HL-60) were originally created in our laboratory from U937 and HL-60 cells, respectively [7]. Prior to specimen collection from patients, the study was approved by the institutional review board of Tokyo Medical University (IRB no. 1974), and written informed consent was obtained from the patients in accordance with the Declaration of Helsinki.

The peripheral blood samples with >80% blasts of leukemia cells were collected from five patients with AML that developed from MDS-acquired AZA resistance. The clinical characters are described in the Supplementary Table 1. Leukemia cells from patients were isolated using Lymphosepar I (Immuno-Biological Laboratories, Fujioka, Japan). CD34-posistive cells were obtained from peripheral blood of a healthy donor treated with G-CSF. After isolation of mononuclear cells using Lymphosepar I, CD34-positive cells were purified using CD34 MicroBead Kit UltraPure (Miltenyi Biotech, Gladbach, Germany) and autoMACS Pro Separator (Miltenyi Biotech). In flow cytometric analysis, the purity of CD34-positive cells was >98% and nearly 80% of them were CD38-negative.

AZA and DAC were purchased from Wako Pure Chemical Industries (Osaka, Japan) and teriflunomide from Selleck Chemicals (Houston, TX, USA). The anti-DNMT1 antibody 4H80, the anti-DNMT3A antibody H-295, and the anti-β-ACTIN antibody C4 were purchased from Santa Cruz Biotechnology (Dallas, TX, USA). The secondary antibodies, namely horseradish peroxidase (HRP)-labeled anti-mouse IgG antibody and HRP-labeled anti-rabbit IgG antibody, were purchased from GE Healthcare (Buckinghamshire, UK).

### Cell culture and chemical reagent treatment

U937, HL-60, R-U937, and R-HL-60 cells and leukemia cells from patients were incubated in RPMI1640 medium (Life Technologies Inc., Carlsbad, CA, USA) including 10% inactivated fetal bovine serum and 1% penicillin/streptomycin (Life Technologies). CD34-positive cells from a healthy donor was cultured in Hematopoietic Progenitor Expansion Medium DXF (PromoCell, Heidelberg, Germany) supplemented with Cytokine Mix E (PromoCell). For treatment with reagents, cells were collected by centrifugation and suspended at 1 × 10^5^ cells/ml in fresh medium with the agents or 0.01% DMSO as the vehicle. Cell viability was measured using the Cell Counting Kit-8 (Dojindo, Kumamoto, Japan) as previously described [25]. CI 25 was calculated using Chou-Talalay method [14]. For continuous treatment, 5 μM AZA and/or 1 μM teriflunomide was added to the U937 cells or HL-60 cells suspended in the fresh medium at 1 × 10^5^ cells/ml at day 0. Then at days 3, 7, 10, 14, 17, 21, 24, 28, 31, 35, 38, 42, and 49, culture medium was changed to fresh medium with the added agents and cell viability was measured using the Cell Counting Kit-8.

### Measurement of pyrimidine salvage activity

Pyrimidine salvage activity was estimated based on the availability of EU. Cells were incubated in the medium with EU for 3 h after treatment with teriflunomide for 72 h. EU incorporated in RNA was labeled with Alexa Fluor 488 using the Click-It RNA Alexa Fluor 488 Imaging Kit (Thermo Fisher Scientific, Waltham, MA, USA). The signal for EU was measured using flow cytometric measurements performed on a BD Accuri C6 Flow Cytometer (BD Bioscience, Franklin Lakes, NJ, USA). A 488-nm blue laser was used for excitation, and the signal was detected by the FL1 channel (533 nm). The signals of 30,000 events were obtained. Analyses of the obtained data were performed using C6 software version 1.0 (BD Biosciences).

### Detection of apoptosis

The FITC Annexin V Apoptosis Detection Kit I (BD Bioscience) was used. Cell lines treated with the reagents for 4 days were suspended in binding buffer and incubated with FITC-labeled annexin V and propidium iodide in the dark.

Flow cytometric measurements were performed on a BD Accuri C6 Flow Cytometer (BD Biosciences). A 488-nm blue laser was used for excitation, and signals were detected using the FL1 channel (533 nm) for FITC and the FL2 channel (585 nm) for propidium iodide. The signals of 30,000 events were obtained. Analyses of the obtained data were performed using C6 software version 1.0 (BD Biosciences).

### Western blotting

Western blotting was performed as previously described [25]. Briefly, the membranes were probed with antibodies directed against DNMT1 (1:200), DNMT3A (1:200), or β-ACTIN (1:500) and then treated with the appropriate secondary antibodies.

### Chromatin immunoprecipitation (ChIP) assay

ChIP assay was performed using ChIP-IT Express kit (Active Motif, Carlsbad, CA, USA) in accordance with the manufacturer’s protocols. Briefly, the chromatin was extracted from 1 × 10^7^ formalin-fixed cells and chromatin including 15 μg DNA was immunoprecipitated using 2 μg anti-DNMT1 antibody or anti-DNMT3A antibody. For negative control, mouse non-specific IgG was used. The amount of precipitated DNA was measured using NanoDrop 1000 (Thermo Fisher Scientific).

### Quantitative RT-PCR

Quantitative RT-PCR was performed as previously described [26]. TaqMan gene expression assays (Life Technologies) were used for *UCK1* (Hs01075618_m1) and *UCK2* (Hs00367072_m1). The TaqMan Pre-Developed Assay Reagent (Life Technologies) was used for *ACTB*. The relative expression level of each gene to the *ACTB* expression level was determined by the ΔCT method.

### Statistical analysis

For statistical analyses, two-way ANOVA followed by the *t*-test were performed using GraphPad PRISM 6 software (GraphPad Software Inc., La Jolla, CA, USA); *p* < 0.05 was considered significant. Data are shown as mean ± SD in the figures, and they represent the results obtained from three independent experiments.

## SUPPLEMENTARY MATERIALS FIGURE AND TABLE



## Abbreviations

AZA: azacytidine
UCK: uridine cytidine kinase
DNMT: DNA methyltransferase
MDS: myelodysplastic syndromes
AML: acute myeloid leukemia
