# Supplementary material for: Teriflunomide restores 5-azacytidine sensitivity via activation of pyrimidine salvage in 5-azacytidine-resistant leukemia cells
Source: Oncotarget. 2017 Jul 22;8(41):69906–15. doi: 10.18632/oncotarget.19436 (PMC5642525; doi:10.18632/oncotarget.19436)
Supplement: Supplementary file 1 [file oncotarget-08-69906-s001.pdf]

Teriflunomide restores 5-azacytidine sensitivity via activation of pyrimidine salvage in 5-azacytidine-resistant leukemia cells

SUPPLEMENTARY MATERIALS

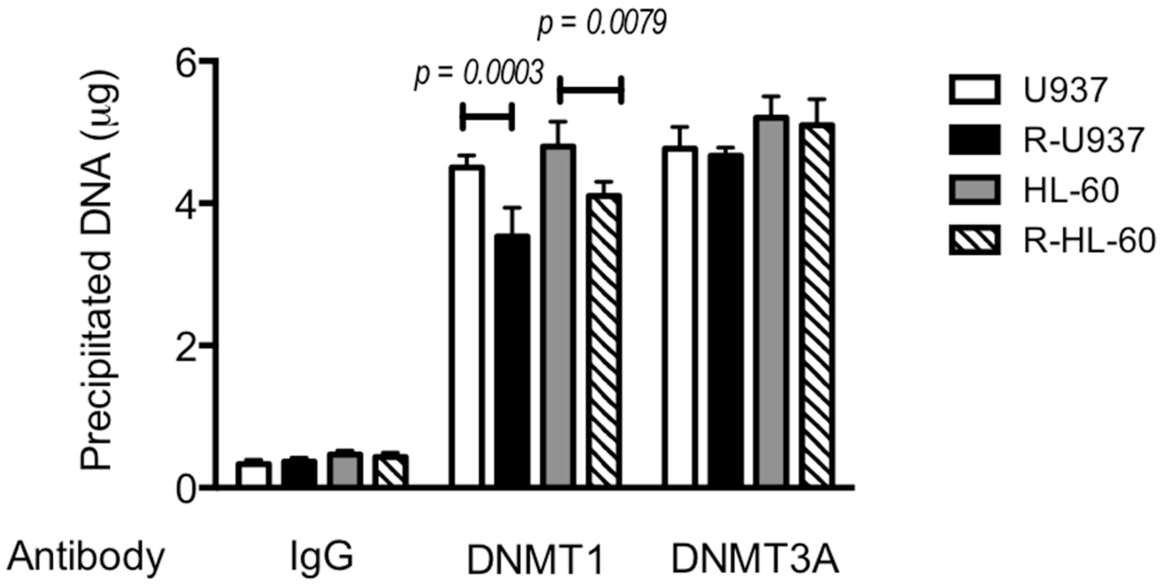

Supplementary Figure 1: The DNA amount precipitated in the ChIP assay using anti-DNMT1 antibody and anti-DNMT3A antibody. Non-specific mouse IgG was used as negative control. DNA amount precipitated by anti-DNMT1 antibody from AZA-resistant cells was significantly less than those from AZA-sensitive counterparts.

Supplementary Table 1: The clinical characters of patients

Patient	Gender	Age	Diagnosis	Number of AZA course	Cytogenetics	Best response
1	Male	77	RCMD overt AML	15	Complex	HI
2	Male	75	CMML overt AML	6	47, XY, +8	SD
3	Male	63	RAEB2 overt AML	14	Complex	HI
4	Male	61	RAEB2 overt AML	7	46, XY, del(20) (q11)	SD
5	Female	73	RAEB2 overt AML	27	45, XX,-7	HI

The acquisition of AZA resistance was judged by the progression of desease. Cytogenetics were investigated at the time point of diagnosis of AML. RCMD: refractory cytopenia with multilinage dysplasia, CMML: chronic myelomonocytic leukemia, RAEB2: refractory anemia with excess blats 2, AML: acute myeloid leukemia, HI: hematological improvement, SD: stable desease.
